# Bioleaching of Typical Electronic Waste—Printed Circuit Boards (WPCBs): A Short Review

**DOI:** 10.3390/ijerph19127508

**Published:** 2022-06-19

**Authors:** Xiaosheng Ji, Mindong Yang, Anping Wan, Shaoqi Yu, Zhitong Yao

**Affiliations:** 1 Cryogenic Center, Zhejiang University City College, Hangzhou 310015, China; jixiaoshen@zju.edu.cn (X.J.); wanap@zucc.edu.cn (A.W.); 2 China Energy Engineering Group Guangdong Electric Power Design Institute Co., Ltd., Guangzhou 510663, China; yangmindong@gedi.com.cn; 3 College of Biosystems Engineering and Food Science, Zhejiang University, Hangzhou 310058, China; yushaoqi@zju.edu.cn; 4 College of Materials and Environmental Engineering, Hangzhou Dianzi University, Hangzhou 310018, China

**Keywords:** waste printed circuit boards, electronic waste, sulfur-oxidizing bacteria, biometallurgy, bioleaching mechanism

## Abstract

The rapid pace of innovations and the frequency of replacement of electrical and electronic equipment has made waste printed circuit boards (WPCB) one of the fastest growing waste streams. The frequency of replacement of equipment can be caused by a limited time of proper functioning and increasing malfunctions. Resource utilization of WPCBs have become some of the most profitable companies in the recycling industry. To facilitate WPCB recycling, several advanced technologies such as pyrometallurgy, hydrometallurgy and biometallurgy have been developed. Bioleaching uses naturally occurring microorganisms and their metabolic products to recover valuable metals, which is a promising technology due to its cost-effectiveness, environmental friendliness, and sustainability. However, there is sparse comprehensive research on WPCB bioleaching. Therefore, in this work, a short review was conducted from the perspective of potential microorganisms, bioleaching mechanisms and parameter optimization. Perspectives on future research directions are also discussed.

## 1. Introduction

**Figure 1:**
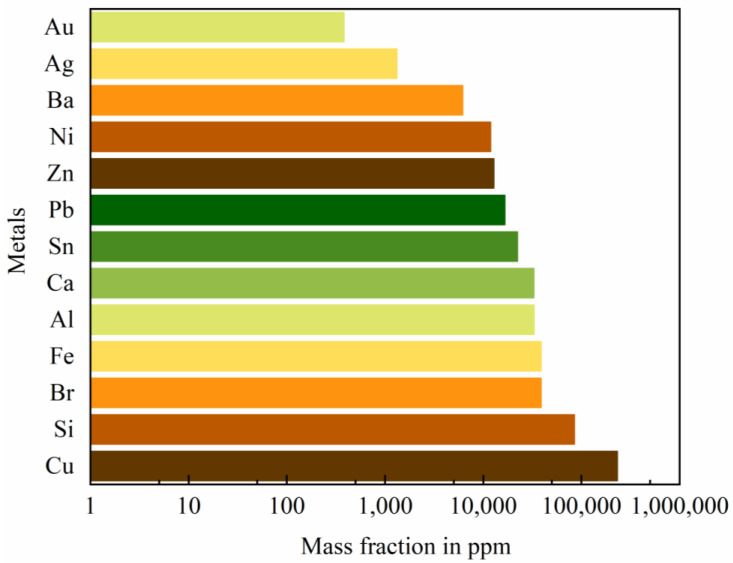
Metal component in WPCBs [6].

Rapid technological innovation has greatly shortened the life cycle of electronic products [1,2]. The massive generation of waste electrical and electronic equipment (WEEE) has displayed exponential growth with a rate of more than 3–5% annually. In 2016, the global amount of electronic waste, so called e-waste, was approximately 44.7 million metric tons (Mt) and has reached 57 Mt in 2021 [3,4]. Printed circuit boards (PCBs) are a major and critical constituent of electrical and electronic equipment [5] and the resultant waste PCBs (WPCBs) account for approximately 3–6 wt.% of total e-waste [6,7]. These WPCBs contain various precious metals, such as gold (Au), silver (Ag) and palladium (Pd) with concentrations tens or hundreds of times larger than than the natural ores [8,9] and have been regarded as an “urban mine” (Figure 1). According to the content of Au, the WPCBs could be divided into low (100 g/t), medium (100–400 g/t), and high (400 g/t) grades [10]. Their net present value varies from 6.8 million € for medium-grade ones to 63.0 million € for high-grade ones [11].

In the past few decades, landfilling, combustion and informal processing activities [12], such as acid stripping and open burning have led to significant environmental pollution. Oloruntoba et al. [13] assessed the status of polybrominated diphenyl ethers (PBDEs) contamination at e-waste dumpsites in Lagos, Nigeria. PBDE levels across the soil profile (0–45 cm depth) showed an increase in PBDE accumulation in the topsoil and migration into the sub-soil. Sediments and rainwater ponds around the dumpsites were found to be contaminated as well. Abubakar et al. [14] studied the heavy metal concentrations, in relation to threshold values, and assessments of risk for noncarcinogenic and cancer risk threat. Pb had the highest mean concentration of 0.0693 ppm, Cu 0.0525 parts per million (ppm), and Cd 0.0042 ppm. The informal e-waste burning had resulted in the substantially high levels of air pollution identified at the treatment points and in turn posed a threat to the environment and public health. Recently, many advanced technologies have been developed to facilitate metal recovery from WPCBs, including pyrometallurgy, hydrometallurgy, physico-mechanical separation, electrolysis, supercritical fluid, and bioleaching (Table 1). Among them, the pyrometallurgical processes are generally operated at 300–900 °C [15,16] and have the disadvantage of high energy consumption and expensive capital investment [17]. Hydrometallurgical processes use cyanide, halide, thiourea and thiosulfate to recover metals [18], consuming large amounts of chemical reagents as well as producing a large volume of effluents. Mechanical beneficiation operations such as gravity air classifiers, eddy current separation, and magnetic separation have been widely used in e-waste recycling plants worldwide. However, the recovered metals are mixed, and need be refined [19].

**Figure 2:**
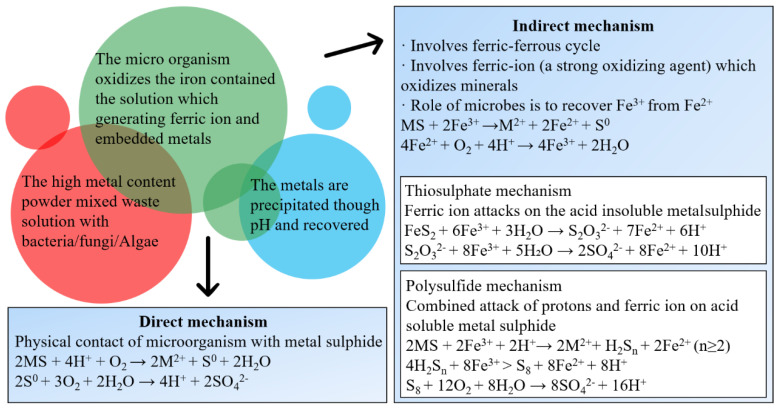
Bioleaching mechanisms of metals from WPCBs [25].

Bioleaching uses a variety of microorganisms, including chemolithotrophic prokaryotes, heterotrophic bacteria and fungi to mobilize metals from WPCBs based on the three following mechanisms (Figure 2). (1) Transformation of organic or inorganic acids (protons); (2) Oxidation and reduction reactions; and (3) Excretion of complexing agents [38]. It overcomes the problems of high energy consumption, serious environmental pollution and complex operation and thus has been regarded as a promising method for metals recovery [39]. Recently, different leaching techniques have been developed. In laboratory-scale investigations, percolator leaching, submerged leaching and column leaching are employed. For industrial-scale purposes, dump leaching, heap leaching, underground leaching and tank leaching are applied [40]. Kumar et al. [41] used *Pseudomonas balearica SAE1* to achieve the dissolution of Au and Ag, resulting in a recovery of 68.5% Au, and 33.8% Ag, at pH of 9.0, pulp density of 10 g/L, temperature of 30 °C, and glycine concentration of 5 g/L. Zhou et al. [42] proposed biological detoxification and comprehensive utilization of non-metal residue from waste copper clad laminate. The leaching rate of metal was close to 100% and was 8.7% higher than that by dilute sulfuric acid leaching. It further demonstrated the potential of microorganisms and the feasibility of the bioleaching approach. The bioleaching of WPCBs has been attempted however, there are sparse systematic studies on this topic. The bioleaching mechanism and optimization of leaching conditions are not clear, and need be further probed. Therefore, a short review was conducted from the perspective of potential microorganisms, bioleaching mechanism and parameters optimization. The perspectives on future research directions were also discussed. Promotion of experimental scale studies to a larger scale in practice is expected to speed up e-waste recycling and result in zero waste buildup in China.

## 2. Microorganisms

**Figure 3:**
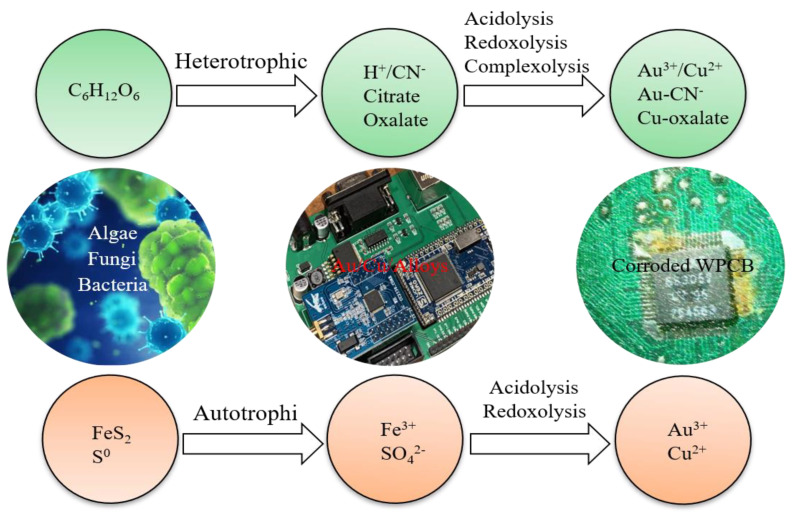
Heterotrophic and autotrophic bioleaching of WPCBs [26].

Microbes were found to have the ability to extract metals a long time ago, therefore microbial technology has been more developed. It has long been noticed that initially microorganisms were only used to extract metals from mine resources. The use of microbial interactions on mineral substances is gaining practical significance. Leaching metals from ores makes it possible to obtain solutions, for example copper or uranium, from which these metals can be recovered using hydrometallurgical processes. Microbes also offer the possibility to recover metals from e-waste. In the bioleaching process, the growth of microorganisms usually involves two stages. The reaction of organic sugar and acid with WPCBs takes place in the first stage, while the second—the development of microorganisms [43]. Most often, bioleaching is carried out in an acidic environment, using processes such as the oxidation of sulphur or its reduced compounds to sulfuric acid and the production of, for example, organic acids in oxygen respiratory cycles or as a result of fermentation of carbohydrates. The microorganisms involved in the leaching include not only bacteria (Acidithiobacillus, Thiobacillus), but also fungi (Penicillium/Aspergillus/Fusarium/Alternaria Candida). Iron and sulfur-oxidizing bacteria (e.g., Acidithiobacillus ferrooxidans, Leptospirillum ferrooxidans and Acidithiobacillus thiooxidans) are widely used in bio-hydrometallurgical process [44]. Generally, there are two different ways of heterotrophic and autotrophic bioleaching (Figure 3), and three groups of microorganisms could be microbial candidates applied for WPCB recovery, including chemolithotrophic prokaryotes (e.g., *Acidithiobacillus thiooxidans*, *Acidithiobacillus ferrooxidans*), heterotrophic bacteria (e.g., *Chromobacterium violaceum*, *Pseudomonas* sp., and *Bacillus megaterium*) and fungi (e.g., *Aspergillus niger*, *Penicillium simplicissimum*).

### 2.1. Autotrophic Bioleaching

The chemolithotrophic organisms use atmospheric carbon dioxide as a carbon source and inorganic compounds such as ferrous iron (Fe^2+^), elemental sulfur (S°) and/or reduced sulfur compounds (S_8_, S_2_O_3_^2−^, H_2_S and polysulfide) as an energy source [45]. These characteristics facilitate metal dissolution through a series of bio-oxidants and bioleaching reactions [46]. According to their preferred temperatures, the chemolithotrophic organisms could be classified as mesophiles (28–37 °C), moderate thermophiles (40–60 °C) and thermophiles (60–80 °C). Most of them grow at a low pH of 2.0 or below, and have a high tolerance for heavy metal toxicity [47]. *Acidithiobacillus ferrooxidans* (*A. ferrooxidans*) was the most well-known and extensively studied microorganism in biometallurgical applications. Bai et al. [48] used *A. ferrooxidans* to leach heavy metals from WPCB sludge. The leaching rates of Cu, Ni, and Zn reached 76%, 74%, and 72%, respectively, under the optimized conditions of FeSO_4_·7H_2_O concentration of 60 g/L, initial pH of 0.5, reaction time of 6 days. In addition to *A. ferrooxidans*, other species such as *Acidiferrobacter thiooxydans* (*A. thiooxydans*), *Leptospirillum ferriphilum* (*L. ferriphilum*), *Ferrimicrobium acidiphilum* (*F. acidiphilum*), *Sulfobacillus thermosulfidooxidans* (*S. thermosulfidooxidans*) were also investigated [49]. Ilyas et al. [50] found the selected moderately thermophilic strains of a mixed adapted consortium of acidophilic chemolothotrophic and acidophilic heterotrophic bacteria could recover 80% Zn, 64% Al, 86% Cu and 74% Ni from WPCB after an acid pre-leaching of 27 days and a bioleaching of 280 days. As compared to using single microbial species, the utilization of different types of chemolithotrophic organisms might yield a better result. It is worth noting that WPCBs are alkaline in nature and more acid was needed to neutralize them, so as to maintain an optimum pH for the microorganisms.

### 2.2. Heterotrophic Bioleaching

Heterotrophic bacteria and fungi are the main microorganisms of heterotrophic bioleaching, relying on organic compounds as energy sources in their metabolism. In the growth phase, they secrete different organic acids such as lactic, citric, oxalic and gluconic acids as well as enzymes [51], which could be used in the leaching process. In contrast to chemolithotrophic organisms, they can tolerate a wider range of pH as well as complex metals and are employed for treating moderately alkaline wastes [52]. More importantly, in the case of some types of WPCB that lack metal sulfides and cannot provide a sufficient supply of energy sources, heterotrophic bioleaching is regarded as a more promising method.

#### 2.2.1. Heterotrophic Bacteria

Heterotrophic bacteria produce acids during bacterial leaching. The byproducts could be utilized to dissolve metals. Cyanogenic bacteria that produce CN are considered the most typical ones. According to the report [53], the first acidophilic heterotrophic bacterium which is indigenous and active in mineral leaching environments was isolated and characterized some 40 years after the iron/sulfur-oxidizing chemolithotroph *T. ferrooxidans* and 70 years after the sulfur-oxidizing acidophile *T. thiooxidans*. After that, more active metal-solubilizing bacteria were isolated and characterized. Various *Pseudomonas* species were used in the bioleaching of Cu, Au, Ag, Pt, and Zn. *Chromobacterium violaceum* was used in the bioleaching of Au from high-grade cell phone WPCBs, reaching a recovery tate of 10.8% Au in 8 days [54].

#### 2.2.2. Fungi

Fungi produce a large amount of organic complexing agents, including citric acid, tartaric acid, oxalic acid, and even carboxylic acids, inducing the solubilization of metals from WPCBs by regulating redox potential and acidity during the fungal bioleaching process, and acidolysis, and redoxolysis mechanisms have been described [55]. Typically, this process is carried out at a relatively higher pH (9.0–10.5) and the fungi are able to adapt to high pulp densities of 10% (*w*/*v*) [56].

Fungal species like *Penicillium chrysogenum*, *Aspergillus niger* and yeast have been thoroughly investigated for bioleaching of metals from solid industrial wastes [57]. According to recent studies, the *Penicillium chrysogenum* strain *KBS3* achieved a maximum solubilization of nickel (55%), copper (67%), magnesium (69%), cobalt (60%), and zinc (65%) from mine tailings [58]. A study was carried out on *Aspergillus niger* in the bioleaching of metals from fly ash [59]. A recovery rate of 56.1% for Cu, 15.7% for Al, 20.5 % for Pb, 49.5% for Zn and 8.1% for Sn were achieved.

It is worth mentioning that the metals in WPCBs could be extracted by bioleaching, however, a significant amount of nonmetallic fraction is left out and remains of great environmental concern. Numerous attempts have revealed that the microorganisms not only have the ability for metal extraction but also are promising for the degradation of plastics. An increasing number of isolates of bacterial (e.g., *Bacillus*, *Rhodococcus*), fungal (e.g., *Aspergillus*, *Penicillium*), and bacterial consortia (e.g., *Souda* and *Agios*, *Bacillus cereus* and *Bacillus sphaericus*) with degradation properties and effects on organic compounds have been identified [60]. Senophiyah-Mary et al. [61] revealed that the microbes used for bioleaching had potential for deteriorating plastics, particularly with the assistance of sunlight, UV, etc.

## 3. Bioleaching Mechanisms

**Figure 4:**
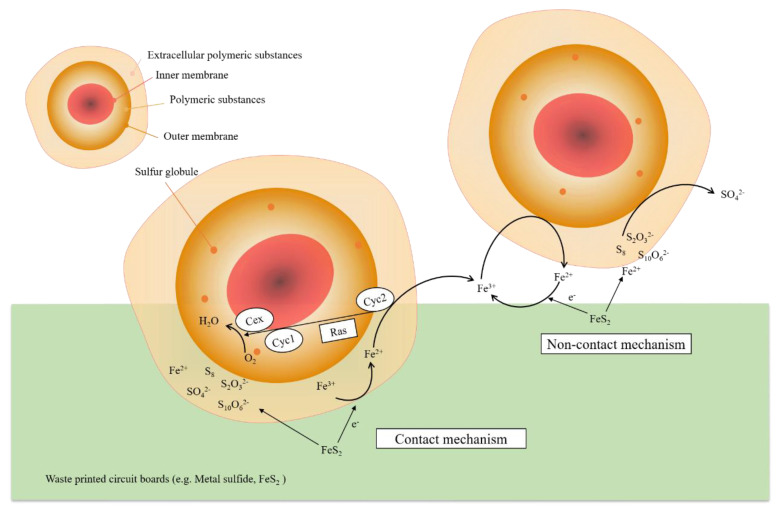
Critical steps in contact and non-contact mechanisms [28].

It should be noted that the mechanisms of microbial interaction are not very clear. The stages of contact and non-contact mechanisms (which are currently known) are presented in Figure 4. For A. ferrooxidans, the main mechanism is indirect, where metals are dissolved through the prior oxidation of ferrous ions. In the case of A. thiooxidans, conversion of metals into a soluble state is mostly done by sulfuric acid production [62]. According to literature sources, in some cases contact bioleaching dominates over the non-contact one, influencing a larger metal recovery process. Mishra et al. [41] explained that it was mainly because the direct mechanism involved direct physical contact of microorganisms with target material surfaces.

### 3.1. Contact Bioleaching

In contact bioleaching, there is physical contact of the microorganisms with metal sulfide (e.g., Pyrite, FeS_2_). Metal solubilization takes place when bacteria such as *A. ferrooxidans* oxidize the metal sulfides and directly obtain electrons from the reduced materials. When the Fe^3+^ from the extracellular polymeric substances (EPS) layer accepts an electron, it will be reduced to Fe^2+^ and diffuse towards the outer membrane, where the ion may be re-oxidized to Fe^3+^ again. The hydrolytic reaction of Fe^3+^ may also be inhibited in acid conditions.

S^0^ + O_2_ + H_2_O → H_2_SO_3_ (1)

2H_2_SO_3_ + O_2_ → 2H_2_SO_4_ (2)

Me + Fe^3+^ + H^+^ + O_2_ → Me^2+^ + Fe^2+^ + H_2_O (3)

4FeS_2_ + 14O_2_ + 4H_2_O → 4FeSO_4_ + 4H_2_SO_4_ (4)

4FeSO_4_ + O_2_ + 2H_2_SO_4_ → 2Fe_2_(SO_4_)_3_ + 2H_2_O (5)

Silva et al. [63] studied the influence of contact mechanisms during Cu bioleaching from WPCBs using a partition system. A reduction of 25% in the extraction was observed, when the contact mechanism was disabled. The bacterial attachment to the WPCB surface was proven to be 4.3 × 10^7^ cells/g, implying that the disabling of bacterial attachment was the only reason for the decrease in the extraction efficiency. To better understand the mineralogical effect of EPS, the micromorphologies of residues after bioleaching were analyzed [64]. Intensive adsorption sites such as rills and micropores were observed on the mineral surface, implying a stronger contact mechanism. Sulfur-oxidizers such as *A. thiooxidans* generally showed a greater dependence on these for their adsorption behavior. Interestingly, the size of the micropores was found to be consistent with the cell size of *A. caldus*.

### 3.2. Non-Contact Bioleaching

In non-contact bioleaching, a ferric-ferrous cycle exists, which involves planktonic (free-living) bacteria oxidizing Fe^2+^ to Fe^3+^, as well as converting sulfur species to sulfuric acid; and the Fe^3+^ ion accepting electrons from metal sulfides and reducing them to Fe^2+^ in turn. In the process, metal solubilization could be described according to the following reaction:

4Fe^2+^ + O_2_ + 4H^+^ → 4Fe^3+^ + 2H_2_O (6)

2Fe^3+^ + Me^0^ → 2Fe^2+^ + Me^2+^ (7)

MeS + Fe_2_(SO_4_)_3_ → MeSO_4_ + 2FeSO_4_ + S^0^ (8)

Gu et al. [65] studied the effect of modified electrode by nitrogen-doped carbon nanotubes in bioleaching copper from WPCB by *A. ferrooxidans*. The results indicated that the Fe^2+^ in the culture medium was used as a nutrient substance by *A. ferrooxidans*, and Fe^2+^ was oxidized to Fe^3+^. The resultant Fe^3+^ would oxidize the copper into a copper ion. Meanwhile, the Fe^3+^ became Fe^2+^.

Cu^0^ + 2Fe^3+^ → Cu^2+^ + 2Fe^2+^ (9)

2Fe^2+^ + O_2_ + 4H^+^ → 2Fe^3+^ + 2H_2_O (10)

2Fe^3+^ + Me^0^ → 2Fe^2+^ + Me^2+^ (11)

Dong et al. [66] recovered vanadium from low grade stone coal using *Bacillus mucilaginosus*. Bioleaching behavior was elucidated through the bacteria-mineral contact leaching and non-contact leaching test. In non-contact bioleaching, there is no need for contact between solids and microorganisms. Only the molecular organic acids in the metabolites interacted with the minerals. The macromolecular compounds in the metabolites cannot form complexes with the metallic elements in the minerals, reducing the opportunities for bacteria to utilize the nutrients in the minerals, thus affecting the growth and metabolism of the bacteria.

## 4. Biochemical Process

Naturally, microorganisms have developed many other processes that influence the biogeochemical cycles of elements (weathering/biooxidation/biotransformation/bioaccumulation/biosorption/bioprecipitation) [67]. Of course, biosorption/bioprecipitation are typical processes that can contribute to the recovery of metals from WPCBs using biological materials. Until now, modern biotechnology has enhanced the ability of metal extraction and optimized performance by understanding the fundamental mechanisms, aimed at economic gain and sustainable development [68].

### 4.1. Biosorption

Biosorption is a passive physico-chemical and metabolically-independent process, based on the ability of living as well as dead microorganisms to utilize a variety of mechanisms, including complexation, chelation, microprecipitation, and microbial reduction, or combinations of them [69]. It does not require large financial outlays, is characterized by high efficiency and minimization of wastes of chemical or biological origin. Moreover, unlike conventional methods (filtration or adsorption on activated carbon), it enables biomass regeneration and metal recovery. In general, the process is mediated through [37]: (1) Extracellular enrichment and precipitation; (2) Exchange and adsorption of cell surfaces; (3) Intracellular transformation. On a cellular scale, metal ions are initiated on the cell surface or active site of biosorbents by the microorganism’s generated ligands through a chelation process [70].

A vast array of biosorbents with potential for metal recovery have been exploited, most of them prepared from natural or waste biomass. A strain of *Penicillium expansum* was used to recover metals, and the resultant product obtained is a highly concentrated solution of lanthanum (up to 390 ppm) and terbium (up to 1520 ppm) [71]. *Chlorella vulgaris* was investigated to recover neodymium from an aqueous solution derived from hard drive disk magnets. The maximum experimental neodymium uptakes at 21, 35 and 50 °C and an initial concentration of 250 mg/L were 126.13, 157.40 and 77.10 mg/g, respectively, at the optimal pH of 5 [71]. Recently, nanomaterials (i.e., nanocellulose structures) and hybrid bio-nanomaterials (assemblies of biological molecules and inorganic nanostructures) with large specific surface areas that could enhance biodegradability and better separation of metal ions, have received attention and are considered the new generation of biosorbents [72].

### 4.2. Bioprecipitation

Bioprecipitation refers to the process of formation of mineral phases (bioprecipitates or biominerals) by the activity of microorganisms. In the process, microorganisms facilitate precipitation by catalyzing oxidative and reductive processes resulting in the precipitation of soluble metals and non-metals [73]. On a cellular scale, bioreduction of metals takes place either by direct contact with the cell surface or through extracellular electron shuttles. Some metal nanoparticles, such as gold elements, were observed depositing on the outer surface of cells. *Desulfovibrio desulfuricans* was one of the microorganisms that was successfully used to recover Au^3+^ as Au^0^ from test solutions and from waste electronic scrap leachate. When processing the aqua regia leachate of Central Processing Units, the *Saccharomyces cerevisiae* were able to rapidly and selectively collect aqueous Au(III) ions from the aqua regia leachate at pH 1.2 within 10 min [74]. By using the sulfate reducing inversed fluidized bed bioreactors, the removal efficiencies of Cu and Zn reached 90% at an initial concentration of 25 mg/L [75].

### 4.3. Parameter Optimization

The bioleaching efficiency is usually not high enough. In most cases, the process is rather time-consuming. In order to improve the efficiency, the bioleaching kinetics were investigated, and the results demonstrated that the interruption in bacterial growth, the formation of precipitates, the toxicity resulting from the increasing level of leached metals, and the increase in the pH of the solutions are all related to the process. In this case, the optimal parameters such as initial pH, temperature, pulp density are considered the critical factors that affect the bioleaching process (Table 2). Understanding their effects well should be beneficial for optimizing their performance.

#### 4.3.1. pH

System pH is an important factor for bioleaching with acidophiles. SOB present in the leaching medium contribute to the system’s acidity which helps in maintaining the system’s pH. This is achieved by the oxidation of sulfur by sulfur-oxidizing bacteria. The optimal pH is 2.0–3.5 for the normal growth of A. ferrooxidans. Crust et al. [76] investigated the effect of pH on the metal dissolution. The leaching of Cu and Ni was seen to be best at an initial pH 1.8, whereas that of Al and Zn had comparable dissolution efficiencies at pH 1.8 and 2.0. At increased pH values (>2 or 2.25), the formation and precipitation of jarosite and/or secondary passivation products was detected, which were unfavorable to the leaching. Gu et al. [65] studied the variation of pH during bioleaching. The pH rose quickly within 5 days and slowly after 7 days, finally reached a steady state. The metabolism of A. ferrooxidans was a process of H^+^ consumption, and the procedure of zero-valent metal turning into metal ions was also a process which could consume H^+^.

#### 4.3.2. Inoculum Volume

Crust et al. [76] investigated the effect of inoculum variation (5–20 (*v*/*v*)) on the metal dissolution. At higher initial inoculum, the costs involved for preparation and consumption of chemicals will be higher but the metal recoveries will be the same as for the 10% (*v*/*v*) initial inoculum. The oxido-reductive potential (ORP) values were nearly similar at higher inoculums indicating a strong oxidizing environment due to the rapid oxidation of Fe^2+^. Fu et al. [77] studied the effect of inoculation volume on metals leaching by *A. ferrooxidans* at 20–35 °C. In the early stage (<50 days), the higher the inoculation number of bacteria was, the higher the leaching rate. On the hand, the number of bacteria on WPCB particles per unit area increased, the contact between bacteria and the active site on the particle surface increased, and metal erosion was accelerated. In the middle-late period (50–80 d), with the growth and reproduction of bacteria and the dissolution of metals, the bacterial number and the ferric-ion concentration in the system with less initial inoculation increased, while the metal-leaching efficiency in the system with more inoculation decreased due to the precipitation of jarosite and the lack of nutrients. Considering the industrial application cost, the optimal inoculation was determined as 5%.

#### 4.3.3. Fe^2+^ Concentration

Iron oxidizing bacteria derive energy by the oxidation of Fe^2+^ ions resulting in the production of biogenic Fe^3+^, which is a powerful oxidizing agent for metals leaching. Therefore, the Fe^2+^ concentration in the medium can affect the metabolism of microbes and the metals leaching rate. Crust et al. [76] investigated the effect of initial Fe^2+^ concentration (1–9 g/L) on the dissolution of Cu, Al, Ni and Zn. With the initial concentration increasing from 7 to 9 g/L, increases of 20% for Cu and 10% for other metals were observed. The maximum ORP values were obtained operating at 9 g/L initial Fe^2+^. Gu et al. [65] investigated the variation of Fe^2+^ concentration during the bioleaching process by *A. ferrooxidans*. The initial Fe^2+^ concentration was 8.09 g/L and it declined obviously in the later bioleaching period. Much research indicates that the lower the Fe^2+^ concentration the final medium has, the better the bioleaching rate the group gets.

#### 4.3.4. Pulp Density

Pulp density also affects the recovery process due to the higher concentration of toxic materials and the change in pH. Garg et al. [78] used mixed microbial consortia of iron and sulfur-oxidizing microorganisms to dispose of waste mobile phone WPCBs for batch bioleaching at varying pulp densities of 7%, 10% and 15% (*w*/*v*). The Ni recovery was 20.39% and 47.9% at a pulp density of 7% and 15%. Crust et al. [76] studied the effect of pulp density in the range 2.5% to 15% (*w*/*v*). The dissolution of Cu was higher at 2.5% (*w*/*v*), whereas the dissolution of Al, Zn and Ni were higher at 10% (*w*/*v*). The dissolution of Cu at 10% PD was 91%. Considering dissolution of all targeted metals, 10% (*w*/*v*) was the best in order to achieve maximum metal recoveries. The increasing solid content had a significant effect on the pH changes of the bioreactor. At ≤10% (*w*/*v*), the diffusion of O_2_ and/or CO_2_ is efficient for oxidation of Fe^2+^, contributing towards higher metal dissolution.

#### 4.3.5. Particle Size

A mechanical activation was discovered to trigger physicochemical changes in solid materials, such as structural defects, phase transformations, and amorphization, to improve their leaching activity [79]. A two-step crushing process has been developed, and heat pretreatment technology before the crushing process has been proposed, as well as for reducing particle size and improving the breakage and liberation effects of WPCBs [80]. During the crushing stage, it should be noted that the micro-cracks produced were beneficial for the growth of bacteria, thereby enhancing the bioleaching efficiency of copper [81].

#### 4.3.6. Catalyst

Studies have shown that graphite can be used as a catalyst to increase the bioleaching rate. In the case of sphalerite, graphite has been found to influence microbial populations [82]. Tong et al. [83] investigated the effect of graphite on leaching behavior of WPCBs. In the absence of graphite, the copper leaching rate was 90% after 5 days. Leaching performance increased with the addition of graphite and it reached 100% with 2.5 g/L of graphite. Gu et al. [35] studied the effect of graphene on bioleaching by *Acidithiobacillus ferrooxidans*. The Cu leaching rate increased from 74% to 84% by adding graphene in the culture solution. The transformation between Fe^2+^ and Fe^3+^ was actuated by *A. ferrooxidans* and graphene. The graphene could be recycled and reused by treating with HNO_3_. They also explored the bioleaching efficiency of Cu driven by nitrogen-doped carbon nanotube modified electrodes [65]. It reached 99% and 20% higher than that of the control. The electrical conductivity and specific surface area which the modified electrode had, provided a good platform for the metabolism of *A. ferrooxidans* and the transfer of electrons.

#### 4.3.7. Bioreactor

Crust et al. [76] investigated the optimal values for key parameters at the shake-flask level and tested the feasibility of these optimal conditions in bench-scale bioreactors. Maximum recovery values of 98.1% Cu, 55.9% Al, 79.5% Ni and 66.9% Zn were achieved under the optimum parameters within 8 days in the laboratory-scale experiments. Under the same optimized conditions, 97.3% Cu, 55.8% Al, 79.3% Ni and 66.8% Zn were bioleached in bench-scale reactors.

#### 4.3.8. Hybrid Bioleaching

Even if the above parameters are optimized, the individual work of those microorganisms is still slow, especially compared to pyrometallurgy and hydrometallurgy processes. Therefore, several studies have been carried out on hybrid bioleaching for better bioleaching effects. For example, Sheel and Pant [84] achieved 90% recovery of Au from e-waste by using the combination of ammonium thiosulfate and *Lactobacillus acidophilus*. Sinha et al. [85] developed a novel biorecovery process followed by electrochemical treatment to recover copper, achieving 92.7% Cu recovery. Dolker et al. [86] applied the chemical-biological hybrid systems to the bioleaching process, increasing Li leaching by 25% and cobalt biosorption by 98%. Gomes et al. [87] linked electrodialytic remediation with microbial metabolism, higher mobilization of metals was observed when associating both methods, with higher metal concentrations in both anode and cathode compartments, in particular for Cu and Cr. All of them exhibited greater bioleaching potential than individual cultures.

**Table 2:** Recent research on the bioleaching of WPCB.

Microorganisms	WPCB	R	Leaching Efficiencies	References
*A. ferrooxidans*	Mobile phone PCB with size of 37–150 µm	Stirring rate of 170 rpm, temperature of 30 °C, initial pH of 1, pulp density of 9.25 g/L, Fe^3+^ concentration of 4.17 g/L	Up to 99% Cu and Ni after 55 days	[88]
	Small pieces with size of <15 mm)	Ambient temperature (20–35 °C), WPCB concentration of 5.0% (*w*/*w*), inoculation volume of 5% (*v*/*v*).	95.92% of Cu, 93.53% of Al, 92.58% of Zn, 65.27% of Ni, and 95.33% of Sn	[77]
*A. niger*	Less than 300 mm	Pulp densities of 0.5–20 g/L, stirring rate of 120 rpm and ambient temperature	100% of Zn, 80.39% of Ni and 85.88% of Cu in 30 days.	[89]
*A. Ferrooxidans* and *A. acidophilum*	Particle size of 0.075–1 mm	Pulp density of 7.5 g/L, pH of 2.5, stirring rate of 170 rpm, temperature of 30 °C	96% Cu, 94.5% Zn, 75% Ni, and 74.5% Pb in 18 days	[90]
*A. Ferrooxidans* and *A. Thiooxidans*	Particle size of less than 100 µm	Stirring rate of 180 rpm, temperature of 30 °C, pulp density of 15 g/L	Cu of 86%, Zn of 100% and Ni of 100% after leaching in 25 days	[62]
*A. Ferrooxidans*, *F. acidiphilum*, and *L. ferriphilum*	Desktop-computer motherboards	Stirring rate of 170 rpm, temperature of 45 °C, pH of 1.6, pulp density of 5% (*w*/*v*), concentration of Fe^3+^ of 9 g/L	100% after adding 2.5 g/L graphite in 5 days	[83]
*A. ferrooxidans*, *L. ferrooxidans* and *A. thiooxidans*	Mobile phone PCB with size of 2 × 2 cm^2^	Initial Fe^2+^ concentration of 9 g/L, pulp density of 10% (*w*/*v*), inoculum of 10% (*v*/*v*) and initial pH of1.8	97.3% Cu, 55.8% Al, 79.3% Ni and 66.8% Zn in bench-scale reactor.	[76]

## 5. Conclusions and Perspective

Summarizing the peer-reviewed review of the program for bioleaching of used PCBs, the bioleaching process offers benefits in terms of low-cost and environmental friendliness and also shows promise in the recovery of metals from WPCBs. Chemolithotrophs, bacteria and fungi can be candidates for the microorganisms involved in the bioleaching process. The chemolithotrophs are ferrous and/or reduced sulfur oxidizers, thriving at a low pH, while heterotrophic bacteria and fungi are considered as organotrophs and tolerate a wider range of pH. Their contact and non-contact mechanisms were analyzed, as well as the biochemical processes of biosorption and bioprecipitation, and it was found that contact leaching played as greater role in this process. As shown by the scientific research, microorganisms adapt to various pH conditions, raise the temperature and density of the pulp in the appropriate range, mechanical activation can strengthen and improve their leaching efficiency. However, there is still much room for improvement in bioleaching and further research is required in industrial applications.

Firstly, most microorganisms have a shortage of time-consuming and environmental limitations. Genetic modification could provide the engineered microorganisms with higher efficiency and rapid adaption to environmental changes.

Secondly, hybrid bioleaching should be developed, and the fundamental mechanisms need to be further probed since some remain uncertain. Moreover, the e-waste usually contains lots of complex materials that might have negative effects on microorganisms’ growth and hinder their metabolism. The collection and classification of different types of waste are key processes for providing pure raw materials, and the pretreatment of WPCBs, such as dismantling and shredding, is an important process for removing toxic components and improving bioleaching efficiency.

Lastly, the bioleaching conditions should be optimized as they affect the biological activities of microorganisms. Novel bioreactors should be built, such as light induced systems and energy harvesting systems [91,92]. The addition of various catalysts also needs to be tested, which might help improve bioleaching efficiency.

## Figures and Tables

**Table 1:** The comparison of different technologies of WPCB treatment.

Technologies	Advantages	Disadvantages	References
Physico-mechanical separation	Simple operation, good economic benefit, and small environmental pollution.	Recovered metals need to be refined. Non-metallic fractions are generated, and volume reduction is not significant.	[20,21,22]
Pyrometallurgy	Significant volume reduction, high treatment efficiency.	High energy consumption and expensive capital investment.	[23,24,25]
Electrolysis	Simple flow sheet, low energy consumption, high output and low environmental pollution.	WPCB must be pretreated, i.e., pre-leaching or supercritical water oxidation.	[26,27,28]
Hydrometallurgy	Short process, high efficiency.	Consumes large amounts of chemical reagents and produces a large volume of effluents.	[29,30,31]
Supercritical fluid	Short process, high efficiency.	High energy consumption and small treatment capacity.	[32,33,34]
Bioleaching	Benefits in terms of low-cost and environmental friendliness	Having a relatively low efficiency and time-consuming.	[35,36,37]

## Data Availability

Not applicable.
